# Reduced orbitofrontal-thalamic functional connectivity related to suicidal ideation in patients with major depressive disorder

**DOI:** 10.1038/s41598-017-15926-0

**Published:** 2017-11-17

**Authors:** Kiwon Kim, Sung-Woo Kim, Woojae Myung, Cheol E. Han, Maurizio Fava, David Mischoulon, George I. Papakostas, Sang Won Seo, Hana Cho, Joon-Kyung Seong, Hong Jin Jeon

**Affiliations:** 1 Department of Psychiatry, Depression Center, Samsung Medical Center, Sungkyunkwan University School of Medicine, Seoul, South Korea; 2 Department of Neuropsychiatry, Seoul National University Bundang Hospital, Seongnam, South Korea; 3 National Institute of Dementia, Seongnam, South Korea; 4 School of Biomedical Engineering, College of Health Science, Korea University, Seoul, South Korea; 5 Department of Psychiatry, CHA Bundang Medical Center, CHA University, Seongnam, South Korea; 6 Department of Electronics and Information Engineering, College of Science & Technology, Korea University, Sejong, South Korea; 7 Depression Clinical and Research Program, Massachusetts General Hospital, Harvard Medical School, Boston, USA; 8 Department of Neurology, Samsung Medical Center, Sungkyunkwan University School of Medicine, Seoul, South Korea; 9 Department of Physiology, Sungkyunkwan University School of Medicine, Samsung Biomedical Research Institute, Suwon, South Korea; 10 Department of Health Sciences & Technology, Department of Medical Device Management & Research, and Department of Clinical Research Design & Evaluation, Samsung Advanced Institute for Health Sciences & Technology (SAIHST), Sungkyunkwan University, Seoul, South Korea

## Abstract

Despite recent developments in neuroimaging, alterations of brain functional connectivity in major depressive disorder (MDD) patients with suicidal ideation are poorly understood. This study investigated specific changes of suicidal ideation in functional connectivity of MDD patients. Whole brain functional connectivity in 46 patients with MDD (23 with suicidal ideation and 23 without) and 36 age- and gender- matched healthy controls were compared using resting-state functional Magnetic Resonance Imaging (fMRI) analyzed with network-based statistics (NBS) and graph-theoretical methods. Decreased functional connectivity in a characterized sub-network was observed in patients with MDD and suicidal ideation (FDR-adjusted *p* < 0.05). The sub-network included the regions of the fronto-thalamic circuits in the left hemisphere. The network measures of the left superior frontal gyrus, pars orbitalis (*r* = −0.40, *p* = 0.009), left thalamus (*r* = −0.41, *p* = 0.009), and right thalamus (*r* = −0.51, *p* = −0.002) were shown, through graph theoretical analysis, to be significantly negatively correlated with severity of suicidal ideation. The reduced functional connectivity in left orbitofrontal-both thalamic regions with suicidal ideation in MDD were inversely proportional to the severity of suicidality independent from depression severity. These findings suggest problems with decision-making and information integration in MDD patients with suicidal ideation.

## Introduction

Every 40 seconds, one individual dies from suicide^1^ while suicide accounts for 1.4% of all deaths, making it the 15th leading cause of death globally^2^. And major depressive disorder (MDD) is the most researched associative factor with suicide, and is noted in 60% of psychological autopsy reports^3^. Fifty eight percent of patients with a current MDD episode report suicidal ideation, with 15% attempting suicide^4^.

Not every suicide attempt is fatal, and suicidal ideation does not always lead to suicide. A previous suicide attempt may be the most important predictor of completed suicide^5,6^. Still, 86% of suicides occur in people with a low risk of suicidal ideation, suggesting that a suicide attempt over a specific period may not accurately reflect the risk of imminent suicidality, a prime target to prevent suicide^7^. It is important to differentiate between suicide attempt and suicidal ideation.

Suicidal ideation is a distinct phenomenon that is not equivalent to depression severity indicators, other dimensions with underlying biology, impact on disability, and risk factors^8,9^. MDD accompanying suicidal ideation is related to a higher rate of previous suicide attempts^10^, poorer treatment response^11^, and is thought to have different neuropsychological correlates that discriminate it from MDD without suicidal ideation^12^. Measuring severity of both depression and suicidal ideation in MDD patients with suicidal ideation may help determine whether severe depression status predicts depression with suicidal ideation. Considering that severity and intensity of suicidal ideation can fluctuate over time, vary according to biological, psychological, and environmental factors^13,14^, suicidal ideation should be assessed as a continuous measurement, and real-time neuroimaging could be a promising approach to examine neural network alterations as correlates of the degree and intensity of suicidal ideation^15^.

Neuroimaging studies have demonstrated that several areas of the brain are associated with suicide attempts at a neuroanatomical level, involving the dorsolateral and orbitofrontal cortex^16,17^. But few studies have focused on MDD with suicidal ideation, and these studies addressed only verbal fluency with functional near-infrared spectroscopy^18^ and cognitive control with fMRI^19^. Few researches reported changes in the fronto-limbic connectivity during the resting state in MDD group with suicidal ideation^20,21^ but further researches should be accumulated to figure out associative characteristics of functional connectivity to with suicidal ideation.

A recent study highlighted the value of considering MDD as a spatiotemporal disturbance of resting activity related to ruminations and enhanced self-focus^22^. In this view, neuroimaging of MDD is a prudent modality to use. MDD patients have been classified into different groups based on dysfunctional connectivity findings from resting state fMRI^23^. Functional connectivity analysis in MDD patients with suicidal ideation would be a useful approach to focus attention on high-risk subgroups^24^.

Graph theory-based connectivity analysis has been successful in investigations of organizational changes in patients by modeling the whole brain as a network^25–27^. Network-based statistics (NBS) analysis^28^ is a useful approach for localizing dysfunctional brain connectivity and is frequently applied to clinical applications^29,30^. Unfortunately, however, there are few data on suicidal ideation in MDD, using resting state functional magnetic resonance imaging (fMRI) with graph theoretical and NBS analyses. Therefore, the aim of this study was to investigate the functional organization of whole-brain networks according to suicidal ideation in MDD patients by NBS analysis and graph theoretical analysis using fMRI. We focused on the comparison between MDD patients with suicidal ideation and patients without suicidal ideation, but we also compared healthy control to rule out the pathologic functional connectivity change coming from depression itself, which can find clear associative functional connectivity in suicidal ideation. We had three hypotheses. First, MDD patients with suicidal ideation will show different functional connectivity in the whole-brain networks compared with MDD patients without suicidal ideation. Second, the degree of the change in functional connectivity in those networks will correlate with the severity of suicidal ideation independently from depression severity. Third, functional connectivity associated with suicidal ideation will differ between MDD patients and healthy subjects.

## Results

### Demographic results

Table 1 shows the demographic and clinical characteristics of the subjects. Forty-six had moderately severe depression, based on initial median Hamilton Depression Scale (HAM-D) score of 19. Seven of the forty-six (15.2%) patients had history of previous suicidal attempt. Patients with suicidal ideation had much higher suicidality outcome measured by the suicidality module of the Mini-International Neuropsychiatric Interview (MINI SM) than patients without suicidal ideation, but five patients without suicidal ideation had a suicidality score of 1, leading to a median score of 3.5 in all MDD patients. Patients with suicidal ideation had a history of more suicide attempts and higher scores with the Scale for Suicide Ideation (SSI). There were no significant differences in gender, age, education, number of episodes, duration of current episode, the Korean version of the Barrett Impulsiveness Scale (BIS) score, HAM-D score, and the Mood Disorder Questionnaire (MDQ) score between the patient groups.

**Table 1:** Clinical characteristics.

**Characteristics**	**Total**	**Suicidal ideation with previous attempt (** ***n*** ** = 7)**	**Suicidal ideation without previous attempt (** ***n*** ** = 16)**	**No suicidal ideation (** ***n*** ** = 23)**	**Healthy controls (** ***n*** ** = 36)**	***P***
Gender, male (%)^*^	14 (17.1%)	1 (14.3%)	2 (12.5%)	2 (8.7%)	9 (25.0%)	0.25
Age, years^†^	56 (50, 62)	47 (42, 56)	57 (54, 65)	52.7 (49, 60)	56.5 (52, 62)	0.091
Education, years^‡^	11.59 ± 4.30	12.29 ± 4.54	11 ± 4.90	11.22 ± 3.79	11.96 ± 4.41	0.815
Number of episodes^§^	2 (1, 2)	3 (2, 5)	2 (1, 2)	1 (1, 2)	—	0.026
Duration of current episode, years^§^	0.55 (0.1, 1.55)	0.6 (0.2, 2.0)	0.3 (0.1, 1.35)	0.5 (0.1, 1.5)	—	0.360
Suicidality score^§^	3.5 (0, 11)	11 (11, 23)	7 (7, 19)	0 (0, 0)	—	< 0.0001
SSI^§^	11 (1.75, 16.5)	21 (12, 25)	15 (10, 21.5)	3 (0, 13)	—	0.001
BIS						
Motor^¶^	15.80 ± 3.93	18.86 ± 4.18	14.31 ± 3.26	15.91 ± 3.86	—	0.035
Attention-cognitive^¶^	15.89 ± 3.16	17.29 ± 3.04	14.63 ± 2.80	16.35 ± 3.24	—	0.049
Non-planning^¶^	20.11 ± 5.16	22.71 ± 5.06	18.38 ± 4.59	20.52 ± 5.34	—	0.162
HAM-D^§^	19 (16.75, 22.25)	18 (16, 19)	21.5 (18.25, 24.50)	18 (16, 20)	—	0.087
HAM-A^¶^	16.94 ± 4.45	16.29 ± 5.77	18.69 ± 5.44	15.91 ± 2.81	—	0.045
MDQ^¶^	4.22 ± 3.34	5.29 ± 2.43	3.69 ± 4.19	4.26 ± 2.94	—	0.262

SSI, Scale for Suicide Ideation; BIS, Barrett impulsiveness scale; HAM-D, Hamilton depression rating score; HAM-A, Hamilton anxiety rating score; MDQ, Mood Disorder Questionnaire.

^*^Fisher’s exact test was used.

^†^Kruskal-Wallis test was used; Data are given as median and interquartile range.

^‡^One-way ANOVA was used; Data are given as mean and standard deviation.

^§^Wilcoxon rank-sum test was used; Data are given as median and interquartile range.

^¶^Student’s *t-*statistics was used; Data are given as mean and standard deviation.

There was also no significant difference in demographic profiles between the two depressive groups and the healthy control group.

### Characterization of sub-network related to suicidal ideation

**Figure 1:**
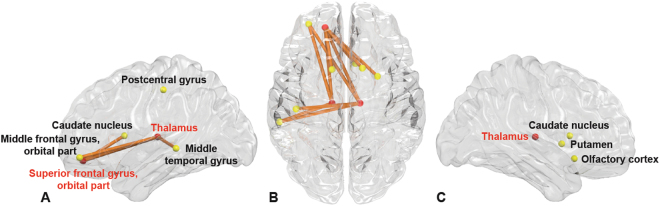
Functional connectivities comparison between MDD patients with and without suicidal ideation. A sub-network shows significantly different functional connectivities between MDD patients with and without suicidal ideation analyzed by network-based statistics. (**A**–**C**) The sub-network mapped on a brain, which represent reduced functional connectivity in MDD patients in the lateral view of the left hemisphere (**A**), the transverse view (**B**), and the lateral view of the right hemisphere (**C**), respectively. The red circles represent the hub regions.

Figure 1 shows a sub-network related with suicidal ideation by comparing functional connectivity between MDD patients with and without suicidal ideation using the NBS analysis. The sub-network includes 11 edges connected with 10 brain regions in both hemispheres (Table 2). These edges represent reduced functional connectivity in MDD patients with suicidal ideation compared with those without suicidal ideation. Among those regions, the orbital part of left superior frontal gyrus, left and right thalamus were defined as hub regions (Fig. 1, denoted by red circles).

**Table 2:** Reduced connectivity in depressive patients with suicidal ideation found using the network-based statistics analysis, compared to depressive patients without ideation.

**Edges**	***t*** **-statistics** ^§^	**Mean functional connectivities**		**Correlation coefficients with clinical measurements** ^†^	
		**Suicidal Ideation (** ***n*** ** = 23)**	**No suicidal ideation (** ***n*** ** = 23)**	**Suicidality score**	**SSI**
Left superior frontal gyrus, pars orbitalis^*^ and left caudate	4.51	0.29	0.52	**−0.56** ^**‡**^	−0.24
Left superior frontal gyrus, pars orbitalis^*^ and right caudate	4.33	0.27	0.48	**−0.47** ^**‡**^	−0.24
Left superior frontal gyrus, pars orbitalis^*^ and right olfactory cortex	4.38	0.19	0.40	**−0.47** ^**‡**^	**−0.34** ^**‡**^
Left superior frontal gyrus, pars orbitalis^*^ and right putamen	4.60	0.11	0.33	**−0.54** ^**‡**^	**−0.40** ^**‡**^
Left thalamus^*^ and left superior frontal gyrus, pars orbitalis^*^	4.89	0.10	0.32	**−0.57** ^**‡**^	**−0.42** ^**‡**^
Left thalamus^*^ and left middle frontal gyrus, pars orbitalis	4.36	0.13	0.35	**−0.50** ^**‡**^	**−0.41** ^**‡**^
Left thalamus^*^ and left middle temporal gyrus	4.47	0.34	0.54	**−0.48** ^**‡**^	**−0.40** ^**‡**^
Right thalamus^*^ and left superior frontal gyrus, pars orbitalis^*^	5.19	0.061	0.29	**−0.58** ^**‡**^	**−0.40** ^**‡**^
Right thalamus^*^ and left middle frontal gyrus, pars orbitalis	4.21	0.084	0.32	**−0.56** ^**‡**^	**−0.37** ^**‡**^
Right thalamus^*^ and left middle temporal gyrus	5.40	0.24	0.51	**−0.54** ^**‡**^	**−0.35** ^**‡**^
Right thalamus^*^ and left postcentral gyrus	4.64	0.20	0.49	**−0.56** ^**‡**^	**−0.52** ^**‡**^

SSI, Scale for Suicide Ideation.

^§^t-statistic from 2-sample *t*-test, (MDD_without_ − MDD_with_).

^*^hub nodes.

^†^Spearman partial correlation coefficients between functional connectivities and Suicidality score/SSI scores in total MDD patients.

^‡^BOLD**:** significant after FDR correction over 11 edges (FDR-adjusted *p*-value < 0.05).

### Correlation analysis between functional connectivity and clinical measurements

The connectivity of all 11 edges of the sub-network were significantly correlated with the suicidality score. In addition, nine edges were also significantly correlated with the SSI scores. The directions of all significant correlations were negative (Table 2). There was no significant correlation between functional connectivity and other clinical measures including Hamilton Anxiety Scale (HAM-A), HAM-D, MDQ, and BIS that represent severity of depression.

### Correlation analysis between network topological measures and clinical measurements

Node strengths, clustering coefficients, and regional efficiencies of all three hub regions were significantly correlated with SSI scores. Furthermore, all these network topological measures were also significantly correlated with suicidality scores, except the node strength of the orbital part of superior frontal gyrus. In addition, the betweenness centrality of the orbital part of superior frontal gyrus was significantly correlated with the BIS motor scores. The directions of the correlation coefficients between the network topological measures and suicidality scores/SSI were negative, while the other was positive (Table 3). There was no significant correlation between the network topological measures and other clinical measures including HAM-A, HAM-D, MDQ, and BIS that represent the severity of depression, which is consistent with the previous findings observed for the edge-level connectivity.

**Table 3:** Significant correlations between clinical variables and connectivity measures in MDD patients (FDR-adjusted p < 0.05).

	**Clinical measurements**	**Correlation coefficients**	**FDR-adjusted** ***p*** **value**
**Node strength**			
Left thalamus	Suicidality score	−0.40	0.007
	SSI	−0.35	0.021
Right thalamus	Suicidality score	−0.47	0.004
	SSI	−0.37	0.021
Left superior frontal gyrus, pars orbitalis	SSI	−0.39	0.021
**Betweenness centrality**			
Left superior frontal gyrus, pars orbitalis	BIS motor	0.38	0.034
**Clustering coefficients**			
Left thalamus	Suicidality score	−0.39	0.016
	SSI	−0.36	0.025
Right thalamus	Suicidality score	−0.44	0.010
	SSI	−0.36	0.025
Left superior frontal gyrus, pars orbitalis	Suicidality score	−0.30	0.049
	SSI	−0.32	0.034
**Regional efficiency**			
Left thalamus	Suicidality score	−0.41	0.009
	SSI	−0.32	0.037
Right thalamus	Suicidality score	−0.51	0.002
	SSI	−0.36	0.026
Left superior frontal gyrus, pars orbitalis	Suicidality score	−0.40	0.009
	SSI	−0.37	0.026

SSI, Scale for Suicide Ideation.

### Comparison between MDD patients and healthy participants

**Figure 2:**
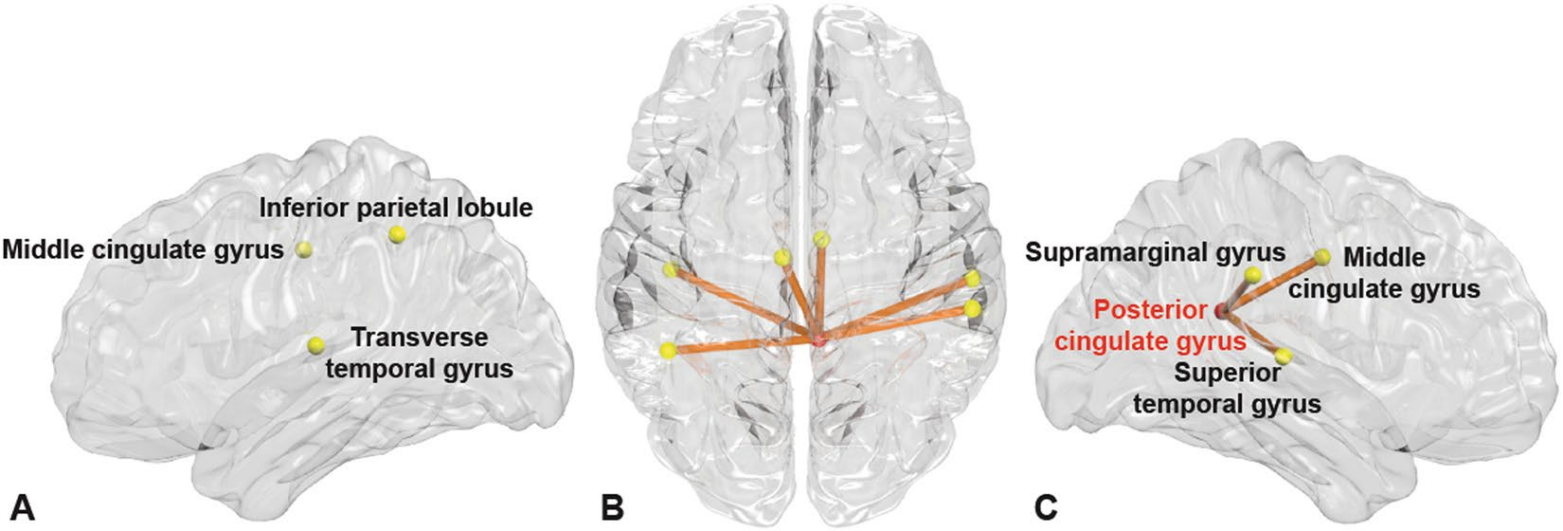
Functional connectivities comparison between healthy participants and MDD patients. A sub-network shows significantly different functional connectivities between healthy participants and MDD patients analyzed by network-based statistics. (**A**–**C**) The sub-network mapped on a brain, which represent reduced functional connectivity in MDD patients in the lateral view of the left hemisphere (**A**), the transverse view (**B**), and the lateral view of the right hemisphere (**C**), respectively. The red circles represent the hub regions.

Figure 2 shows a sub-network related with MDD by comparing functional connectivity between total MDD patients and healthy group using the NBS analysis with same initial threshold and permutation number as the analysis between MDD patients with and without suicidal ideation. The edges in the sub-networks represent reduced functional connectivity in MDD patients compared with the healthy group. The sub-network included six edges connected with seven regions including left and right middle cingulate gyrus, right posterior cingulate gyrus, left inferior parietal gyrus, right supramarginal gyrus, left transverse temporal gyrus, and right superior temporal gyrus. Only the right posterior cingulate gyrus was defined as a hub region (Fig. 2, denoted by red circles).

## Discussion

To the best of our knowledge, this is the first study comparing resting state functional connectivity in whole-brain network between MDD patients with and without suicidal ideation. We observed that a distinct brain network characterized functional connectivity differences identified in patients with suicidal ideation versus patients without suicidal ideation. This network includes the regions of the orbitofrontal cortex, especially the left superior frontal gyrus, pars orbitalis, left middle frontal gyrus, pars orbitalis, and right olfactory cortex. Additionally, the functional networks consisting of the left middle temporal gyrus, left postcentral gyrus, and subcortical regions including both caudate, both thalami, and right putamen were significantly different in MDD patients with suicidal ideation. There was significant correlation between suicide severity and functional connectivity measures in the subnetwork in the NBS analysis. These functional networks were completely distinct from the functional networks distinguished between MDD patients and healthy subjects.

Regions associated with suicidal ideation among MDD patients observed in our study are involved in decision making^31^ and information integration^32^ with self-attribution^33^. The primary regions apparent from NBS analysis were the left superior frontal gyrus, pars orbitalis, and both thalami; the hub regions are presented in Table 2. Figure 1 illustrates the hub regions and decreased edges included in these regions, representing the reduced amount of functional connectivity between left superior frontal gyrus, pars orbitalis, and both thalami according to the groups. The left superior frontal gyrus, pars orbitalis is the upper part of the orbitofrontal cortex and is associated with adaptive cognitive and emotional behaviors like decision-making^31,34^. Another hub region that showed reduced functional connectivity was both thalami, which are in the central area that is involved with transmitting information through the brain, and communication with many associative brain regions and global multifunctional pathways^35^. The thalamus also contributes to homeostatic brain rhythms that function in basic inhibition and coordination^36^. In the mood-related neural network, the thalamus receives strong dopaminergic projections, playing a critical role for information integration, which is responsible for depression and suicidal behavior^37,38^.

Poor decision-making coupled with maladaptive information integration is linked with suicidal ideation or behavior^39,40^. Decision-making represents a higher level of executive function based on information integration, leading to cognitive flexibility and appropriate social judgement with behavioral control^41^. These impairments of cognitive actions have clinical implications for suicide in depression. The present results strengthen the view that the alterations in functional networks involved in decision-making with information integration are associated with suicidal ideation. Care is needed in interpreting these results, given that the study was designed to indirectly investigate the association between decision-making, suicidal ideation, and sub-network alterations due to lack of a decision-making task.

Reduced functional connectivity in fronto-subcortical circuits involving the regions was observed between the left superior frontal gyrus, pars orbitalis, and both caudate; between the left superior frontal gyrus, pars orbitalis, and right putamen; between both thalamus and left middle frontal gyrus, and pars orbitalis; and between the right thalamus and left postcentral gyrus (Fig. 1 and Table 2). Fronto-subcortical circuits are responsible for emotional regulation, executive function, and impulse control; the findings of impaired fronto-subcortical networks are consistent with other studies associated with suicidal behavior beyond their diagnosis^42^. The hub nodes were limited to reduced fronto-thalamic functional connectivity, consistent with previous studies that demonstrated impaired fronto-thalamic circuitry in suicidal MDD patients through DTI^43,44^. Dysfunctions in these circuits may also result in the loss of prefrontal cognitive control through the subcortical area. These disturbances might increase the risk for dysregulation of emotional responsivity and vulnerability to suicidal ideation and behavior.

Presently, the left lateralization related to suicidal ideation was limited to the left middle frontal gyrus, pars orbitalis, left middle temporal gyrus, and left postcentral gyrus. The left middle frontal gyrus and middle temporal gyrus contribute for deductive reasoning, which has been most often observed with left lateralization^45,46^. Along with the left postcentral gyrus, three of these regions provide information from a person’s previous experiences and build mental simulation to make decisions. Deductive reasoning based on previous information and simulation is related to self-attribution, and negative attributional style is associated with suicidality that successfully resolves after treatment^47^. Hence, reduced functional connectivity in the left lateralized middle frontal and temporal networks could be related to suicidal ideation by altering the appropriate deductive reasoning and self-attribution in depressive patients. However, this research was not designed to evaluate asymmetry, and so the findings should be carefully interpreted.

Our correlation study, with network measures and hubs, provides a complementary view of the left superior frontal gyrus, pars orbitalis, and both thalamic regions. We applied graph theoretical analysis and measured the capacity of the overall information segregation, represented as functional connectivity. By identifying the brain’s modular structures, this graph theoretical analysis can detect groups of functionally associated regions responsible for specific functions. This approach (termed connectomics) has been applied to differentiate characteristics of healthy brain networks from other networks with disorders^48,49^.

Node strengths, clustering coefficients, and regional efficiencies displayed significant negative correlations with the severity of suicidal ideation (Table 3), and the betweenness centrality of given nodes was significantly positively correlated with motor impulsivity. Node strength is the sum of weights of links connected to the node and represents the influence of its network^50^. Clustering coefficient is a measurement of local segregation of network and is equivalent to the fraction of a node’s neighbors^50^. Regional efficiency represents the level of intermodular connectivity, which is responsible for communicating information between modules. The betweenness centrality of a given node is defined as the number of shortest paths between any two nodes going through this node, and represents the level of influence on information transformation^51^. These results suggest an impaired function of coordinating brain networks in the left superior frontal gyrus, pars orbitalis, and both thalami in patients with higher suicidal severity, and could be an indicator of pathological changes in this region^44^.

We did not find any significant network measure associated with other depressive symptom severity scales, anxiety scales, and impulsivity scales. In between impulsivity scales, only the motor scale representing impetuous action was significantly associated with reduced functional connectivity in MDD patients with suicidal ideation. The results suggest a possible association with impetuous action in suicidality of MDD and support the previously published association of the BIS motor scale with suicidal ideation in bipolar patients^52^. These negative correlations also highlight reduced orbitofrontal-thalamic functional connectivity as an independent finding related to suicidal ideation, free from other depressive features and impulsivity. However, careful consideration of the findings is needed because of the possibility of false-negative results due to multiple tests and the small sample size.

**Figure 3:**
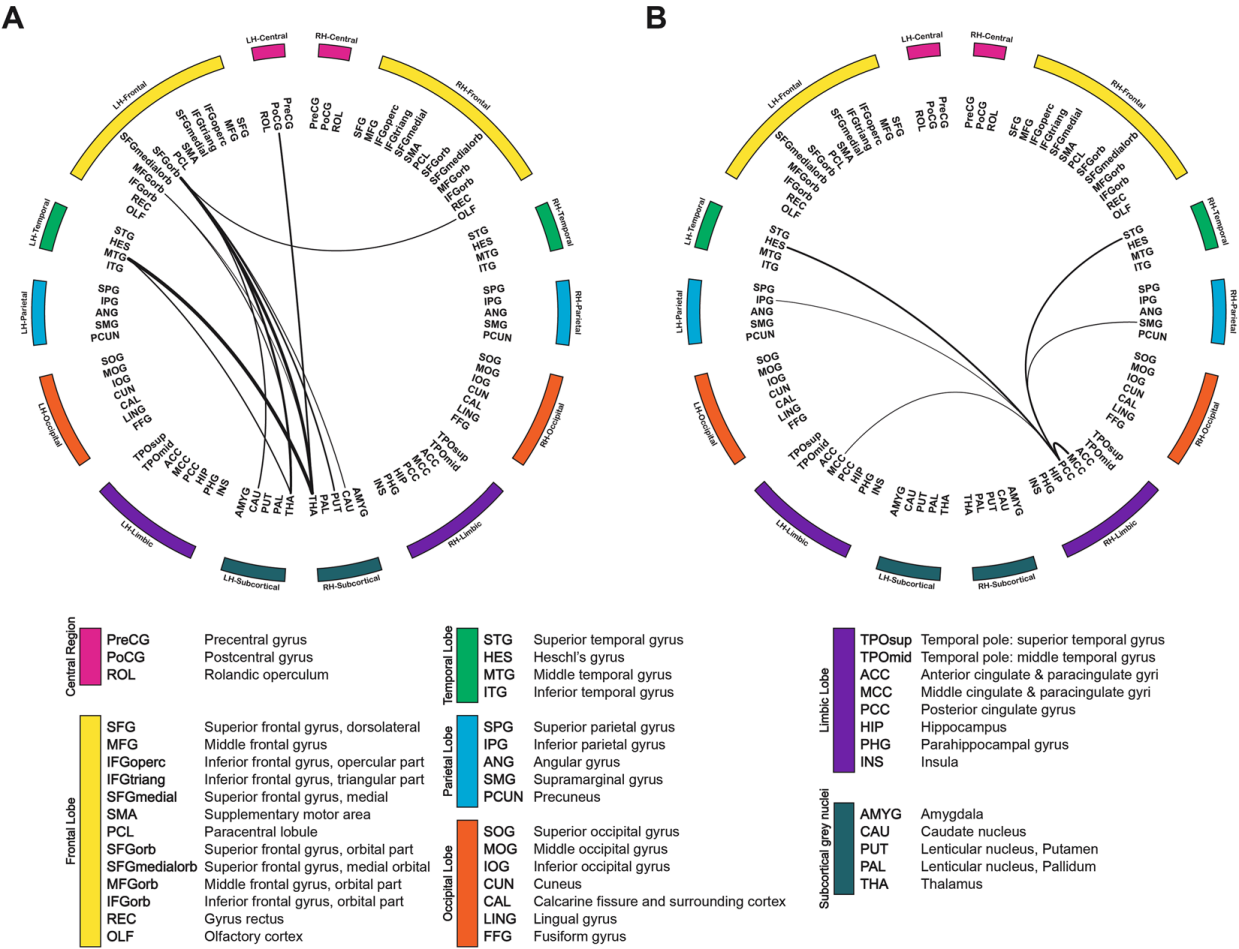
Connectograms showing sub-networks related to suicidal ideation and major depressive disorder. Connectograms showing subnetworks related to suicidal ideation (**A**) and major depressive disorder (**B**). The thickness of connections represents the t-statistics of the network-based statistics test. Between two sub-networks, there is no overlapping brain region or connection, which could indicate neurological changes associated with suicidal ideation independent of depressive status. The classification and abbreviations of brain regions are referred to the references^28^.

Interestingly, no overlapping regions representing significant alterations were evident upon a comparison of MDD patients with healthy controls, and alterations upon comparison of MDD patients with or without suicidal ideation (Fig. 3). Reduced functional connectivity was apparent in the right posterior cingulate gyrus as hub region, both middle cingulate gyri, left inferior parietal gyrus, left transverse temporal gyrus, right supramarginal gyrus, and right superior temporal gyrus in MDD patients compared with healthy controls. These regions are the primary compartment of the default mode network, which engages in autobiographical memory retrieval, envisioning the future, and conceiving the perspectives of others^53^. Other observations that have been noted in meta-analyses are decreased connectivity between posterior DMN and central executive network^54^, especially emphasized in the right hemisphere^55^.

The complete independent functional connectivity evident in this research suggests that the sub-networks are specifically involved in the development of suicidal ideation, and are not associated with depression status or severity of the disorder. Patients with premorbid reduced functional connectivity in the sub-network might be more at risk to develop suicidal ideation when they experience depressive episodes. Another view is that suicidal ideation has its own neurobiological changes that are independent of specific psychiatric disorders. Beyond the diagnosis, two recent studies generalized the discovery, prioritization, validation, and testing of few suicidality related markers across major psychiatric disorders to understand commonalities and differences of suicide^56,57^.

The methodological limitation of our study is the cross-sectional design, which prohibits drawing a conclusion of a causal relationship. Smoking history, as significant risk factor for suicidal behavior^58^ including suicidal ideation^59^, was not evaluated in our study as covariate, but should be considered as an important covariate in further study associated to suicidality. Brain imaging studies that examine superficial phenomena related to suicide-related behavior and more diverse molecular biology techniques for the pathogenesis of suicidal behavior are needed. Interpretation with fMRI data, especially negative findings, should be done carefully, recognizing the potential for signal dropout, geometric distortion, and susceptibility artifacts in the orbitofrontal cortex because of its close proximity to the air-filled sinuses^60,61^.

In conclusion, reduced functional connectivity in orbitofrontal-thalamic regions in MDD patients with suicidal ideation suggests difficulties in decision making and information integration. Reduced functional connectivity correlated only with suicidality, and was independent from severity of depression, anxiety, and impulsivity. The correlation between functional connectivity and suicidal ideation score was significant after controlling the effects of those scores as covariates. This could suggest our finding with pure independent suicidal pathology associated with reduced fronto-thalamic functional connectivity. It could suggest another perspective on suicidal prevention independent from severe depression leading to suicide, but more focused approach on suicidal risk itself. Supplemented evaluation of attribution style and executive function accompanied with neuroimaging focused on default node network are suggested avenues of future research.

## Materials and Methods

### Subjects

Subjects were recruited from April 2011 to April 2013 through the outpatient clinic of the Depression Center of the Samsung Medical Center. These 46 patients comprised 23 MDD patients with suicidal ideation and 23 MDD patients without suicidal ideation who were matched demographically for age, gender, and education year. No patient took any psychotropic medication within two weeks of participation in the study or fluoxetine within four weeks. Inclusion criteria were: age ≥ 18 years, current unipolar major depressive episode as verified by Diagnostic and Statistical Manual of Mental Disorders Fourth Edition (DSM-IV-TR) criteria for MDD^62^, and HAM-D 17-item score ≥16^63^. A board certified psychiatrist evaluated the results of the full version of the MINI^64^, which was repeatedly applied to each patient by another psychologist blinded to the study. Exclusion criteria were: any psychotic disorder (e.g., schizophrenia or delusional disorder), bipolar affective disorder, neurological illness including significant cognitive impairment or Parkinson’s disease, mental retardation, significant medical conditions, epilepsy, history of dependence on alcohol or drugs, personality disorders, or brain damage.

Thirty-six healthy volunteers with no history of psychiatric disease were recruited from advertisements. We excluded participants with a positive family history of a mood disorder. The study protocol was approved by the ethics review board of Samsung Medical Center, Seoul, Korea. And all methods were performed in accordance with the relevant guidelines and regulations approved by the same ethics review board. All subjects were fully informed and consented prior to participation.

### Psychiatric evaluation

From the baseline visit, suicidal ideation was assessed with the suicidality module of the MINI and suicidality score was assessed with total number of points in the suicidality module of the MINI^64^. Subjects replying “yes” to the question “Did you think about suicide during the past one month?” were classified as the ‘suicidal ideation group’ (*n* = 23), and those replying “no” were classified as the ‘no suicidal ideation group’ (*n* = 23). The severity of suicidal ideation was measured by both the suicidality score and the SSI^65^. BIS was applied to evaluate self-report impulsivity^66^. The severity of depression was measured by the 17-item HAM-D^63^. MDQ^67^ was used to assess bipolrarity in depressed patients^68^. The same trained rater applied HAM-D and MDQ.

### Image acquisition

All imaging was performed on a Philips 3.0-T Intera Achieva MRI scanner (Philips Medical Systems, Best, the Netherlands) within one week after the baseline visit. Functional images were obtained using a two-dimensional echo planar imaging-sensitivity encoding (EPI-SENSE) sequence with the following parameters: voxel size 2.86 × 2.89 mm; slice thickness of 4 mm, repetition time (TR) of 3000 ms, echo time (TE) of 35 ms, flip angle of 90°, and matrix size of 220 × 220 pixels. Images were reconstructed to 128 × 128 over a 220 mm field of view. During the scan, all subjects were instructed to rest quietly with their eyes closed and not to fall asleep. A whole-brain three-dimensional fast field echo T1-weighted structural image was acquired with scan parameters: 1 mm sagittal slice thickness, over-contiguous slices with 50% overlap, no gap, TR of 9.9 ms, TE of 4.6 ms, flip angle of 8°, and matrix size of 240 × 240 pixels. Images were reconstructed to 480 × 480 over a 240 mm field of view. All axial sections were acquired parallel to the anterior commissure-posterior commissure line.

### Image preprocessing and network construction

Data from resting state fMRI scans were preprocessed using fMRI Expert Analysis Tool (FEAT) of the FMRIB Software Library (FSL, http://www.fmrib.ox.ac.uk/fsl). Motion correction was carried out using MCFLIRT^69^, and temporal high pass filter using Gaussian-weighted least-squares straight line fitting with cut-off of 126 s was applied^70^. Volumetric regions of interests (ROIs) were defined based on automated anatomical labeling (AAL) atlas, which includes 40 cerebral cortices and five subcortical regions for each hemisphere^71,72^. All time-series signals in each ROI were averaged, so that 90 node-averaged time-series signals in every subject were made.

Resting-state functional networks were constructed by measuring pair-wise similarity between two time-series signals in every ROI using Pearson product-moment correlation coefficients. All correlation coefficients were transformed to z-scores using Fisher r-to-z transformation^48,73–77^. We defined the brain functional networks to have only positive coefficients by discarding functional self- and anti-correlations, which are hard to quantify and interpret the network topological measures^78^.

### Statistical analyses

Clinical and demographic profiles are demonstrated with categorical variables and continuous variables. Categorical variables are depicted with frequencies and proportions. Continuous variables are presented as mean ± standard deviation (SD) or as median and interquartile range, and according to the normality of the distribution, Student’s t test, one-way ANOVA, Wilcoxon rank-sum test, or Kruskal-Wallis test was applied.

NBS was used to detect the significantly different sub-networks between MDD patients with and without suicidal ideation, and between healthy participants and total MDD patients, respectively. NBS is a mass-univariate testing method based on a network component, rather than an individual link, that controls family-wise error rate (FWER)^28^. In the NBS analysis, we first perform two-sample t-test for all edges, resulting 4005 t-statistics for each subject. Then we randomly re-assigned the entire subject sample $N-1$ times, divided into two groups with same sample sizes again, and computed *t*-statistics for all permutations. We computed the maximum component size of sub-networks whose edges had bigger *t*-statistics than a certain initial threshold for all permutations, and formed a null-distribution of maximum component sizes. We checked whether the maximum component size of sub-networks in original set was bigger than those of randomly re-assigned sub-networks at certain significance level. The initial threshold and the number of permutation (*N*) were 4.20 and 10000, respectively.

Hub regions extracted from the sub-networks were identified by comparing MDD patients with and without suicidal ideation. Since hub regions have nodal degrees larger than two standard deviations away from the network mean, they signify the most affected regions. The network topological measures on these hub regions, including nodal strength, regional efficiency, clustering coefficient, and betweenness centrality, were computed as the measures using the Brain Connectivity Toolbox^78^.

In conclusion, correlation analyses were performed between the functional connectivity of the sub-networks and the neuropsychological measures. Correlation tests were also performed between the network topological measures on the hub regions and the neuropsychological measures. For the correlation analyses, correlation coefficients were calculated using Spearman partial correlation after controlling the effects of age, gender, and education years.
